# Recent Advances in Liquid Crystal Polymer-Based Circularly Polarized Luminescent Materials: A Review

**DOI:** 10.3390/polym17141961

**Published:** 2025-07-17

**Authors:** Fa-Feng Xu, Jingzhou Qin, Yu-Wu Zhong, Dandan Gao, Yaping Dong, Haitao Feng

**Affiliations:** 1 Key Laboratory of Green and High-End Utilization of Salt Lake Resources, Qinghai Institute of Salt Lakes, Chinese Academy of Sciences, Xining 810008, China; dyp811@isl.ac.cn; 2 Qinghai Engineering and Technology Research Center of Comprehensive Utilization of Salt Lake Resources, Xining 810008, China; qinjingzhou23@mails.ucas.ac.cn (J.Q.); gaodandan@isl.ac.cn (D.G.); 3 University of Chinese Academy of Sciences, Beijing 101408, China; 4 Institute of Molecular Engineering Plus, College of Chemistry, Fuzhou University, Fuzhou 350108, China

**Keywords:** circularly polarized luminescence, liquid crystal polymer, chirality transfer, construction strategy

## Abstract

Circularly polarized luminescence (CPL) materials have shown great application potential in the fields of three-dimensional displays, bioimaging, and information encryption and decryption. The chirality enhancement of CPL by a physical chiral environment, involving the delivery of structural asymmetry from helical architectures to luminescent molecules through electromagnetic field resonance, represents an innovative approach for constructing high-performance CPL materials. Liquid crystal polymers (LCPs), possessing helical superstructures, show great potential in constructing CPL systems. By modulating the chirality transfer from the helical structural environment of LCPs to luminescent sources via distinct strategies, the CPL properties of LCP-based composites are readily generated and tailored. This review summarizes the newest construction strategies of LCP-based CPL materials and provides a perspective on their emerging applications and future opportunities. This review can deepen our understanding of the fundamentals of chirality transfer and shed light on the development of functional chiral luminescent materials.

## 1. Introduction

Chirality refers to a type of structural asymmetry where a chiral structure cannot be superimposed on its own mirror image. Given the pervasive presence of chirality in the universe, chiral structures emerge across a spectrum of hierarchical levels, encompassing microscopic entities (such as atoms and molecules), mesoscopic constructs (like supermolecules and aggregates), macroscopic entities, and even galactic systems [1,2,3]. For instance, the significance of homochirality for human life is widely recognized; during the evolutionary process, left-handed amino acids and right-handed sugars were selectively favored and ultimately became the fundamental molecular building blocks of the human body [4]. In addition, chirality holds an irreplaceable role in the field of medicine, particularly in drug development. Drugs with specific chirality are effective, whereas their opposite enantiomers may be ineffective or even toxic [5]. In the realm of luminescent materials, chirality is exhibited through distinct polarized luminescence, specifically circularly polarized luminescence (CPL). The existence of actively emissive CPL materials provides a direct and effective way to characterize chirality, avoiding the energy loss (at least 50%) by the physical characterization method via a polarizer and a *λ*/4 waveplate. More importantly, CPL materials build an important bridge for the study of the relationship between the structures and properties of chiral functional materials.

CPL represents the specially fluctuated light emitted from materials, with its electromagnetic vector end traveling on a helical route along its propagation direction [6]. CPL materials have found their vital position in the fields of three-dimensional displays, bioimaging, optical sensing, and information encryption and decryption over the past few decades [7,8,9,10]. To quantify the chirality of circularly polarized lights emitted from a substance, the dissymmetry factor (*g*_lum_) is inducted and defined as *g*_lum_ = 2 (I_L_ − I_R_)/ (I_L_ + I_R_), where I_L_ and I_R_ are the intensities of the left-handed CPL and right-handed CPL, respectively. The maximum of |*g*_lum_| is 2, which represents an optimal and complete emission of left-handed CPL (*g*_lum_ = +2) or right-handed CPL (*g*_lum_ = −2), respectively. The minimum of |*g*_lum_| is 0, indicating non-circularly polarized light is detected. In the field of CPL materials, achieving large dissymmetry factors and high fluorescence quantum yields still remains a big challenge.

**Figure 1:**
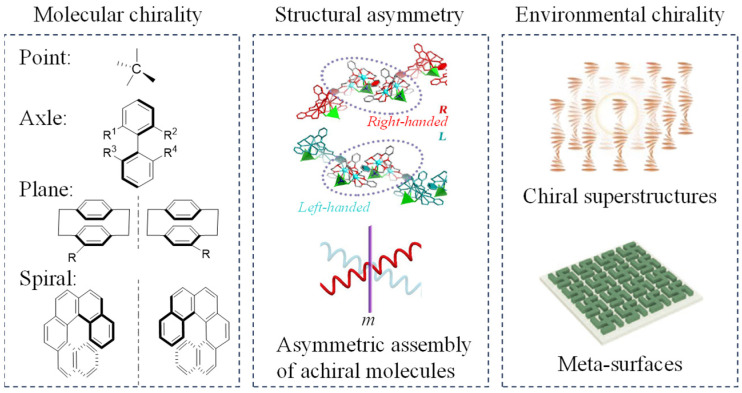
Schematic illustration for three primary scenarios for CPL chirality origin [19,25,26]. Reproduced with permission from [19]. Copyright 2024, Springer Nature. Reproduced with permission from [25]. Copyright 2023, Wiley–VCH. Reproduced with permission from [26]. Copyright 2019, American Chemical Society.

In recent years, a variety of CPL materials have been developed, including organic small molecules [11,12,13,14,15], organometallic compounds [16,17,18], liquid crystals (LCs) [19,20,21], polymers [22], inorganic nanomaterials, and other complex systems [23,24,25]. For these well-developed CPL materials, the origin for their luminescent chirality can be classified into three primary scenarios: (1) the molecular chirality at microscopic level, (2) the structural asymmetry in mesoscopic assemblies, and (3) the influence of mesoscopic environmental chirality (Figure 1). First, molecular chirality stems from those molecules with chiral atoms (C, S), chiral axis, chiral planes, spiral structures, etc., where the inherent molecular chirality and intermolecular chirality induction ability endow the compounds the chiral luminescent property. Second, structural asymmetry arose from assembly process can lead to symmetry breaking in optical properties, that is, differential absorption and emission of chiral light, giving rise to distinct circular dichroism absorption and CPL characteristics of aggregates. Intriguingly, this symmetry breaking in optical properties can even occur when achiral molecules aggregate in an asymmetric manner [26]. Up to now, research on the first two chirality origins (especially the molecular chirality) of CPL materials has been extensively investigated within the realm of materials science. However, the acquired CPL properties of those materials only show limited luminescence chirality, with the |*g*_lum_| values of most CPL materials usually ranging from 10^−4^ to 10^−2^. This is because the inherent chirality at the molecular or aggregate scale is weak, even though the self-assembly process can moderately amplify the chirality of luminescence. There is an urgent need for more efficient and reliable strategies for the development of high-performance CPL materials.

The method of chirality transfer via environmental influence, as the third strategy for generating the chirality of CPL materials, has garnered increasing attention in recent years. Mesoscopic chiral environments that can transfer the environmental chirality to the luminescent sources for generating chiral luminescence are mainly divided into two categories. One is the chiral template structures (such as helix structures from liquid crystals and helical polymers), and the other is the meta-surface structures. Utilizing mesoscale helix-structured materials as templates to conduct the chirality to luminescent sources is an effective approach to obtain high-performance CPL [27,28,29], despite the meta-surface materials that need the relatively hash micro-nano processing conditions. Liquid crystal polymers (LCPs), as one kind of soft materials, are one potential candidate system for achieving large |*g*_lum_| values owing to their inherent helical structures for serving as chiral templates and intrinsic photonic bandgaps for providing an effective chirality transfer path via energy resonance enhancement [30,31,32,33]. In LCPs, liquid crystal molecules, luminescent sources, chiral additives, polymer monomers, initiators, crosslinkers, and so on, play distinct roles in such composite systems. Especially, the liquid crystals in LCPs, including the chiral sematic, cholesteric, and other liquid crystal phases, play a vital and direct part in CPL generation. In addition, the chiral additives can induce the chirality to produce the helical structures for non-chiral liquid crystals, e.g., nematic liquid crystals, and modulate the chirality of chiral liquid crystals, e.g., sematic and cholesteric liquid crystals. The same chirality of the additives and helical structures will enhance the torsional strength and decrease the helical pitch of liquid crystals to amplify the CPL properties, and the opposite chirality will show opposite effects.

In the field of CPL materials, there have already been numerous professional review articles that covered the design, preparation, properties, and potential applications of CPL materials [11,17,34,35,36,37,38,39,40]. It should be noted that although circularly polarized lights can affect liquid crystal molecules in several ways, including medium transmission, selective reflection [41], circular polarization preservation, optical reorientation, and luminescence enhancement, and so on, in this review, we focus on the representative construction strategy for the liquid crystal polymer-based CPL materials and the mechanism of CPL in these material systems; their future development is also discussed. According to the connection between the luminescent source and LCPs, the LCP-based CPL systems are divided into two categories: covalently bonded LCP systems and LCP systems formed by non-covalent bond interactions. The non-covalent interaction formed LCP systems are further subdivided into several subcategories in terms of the construction strategy for distinct luminescent sources, including achiral dyes, aggregate-induced-emission molecules, quantum dots, helical polymers, and so on. It is hoped that this review will provide valuable reference for the construction of new-type high-performance CPL materials.

## 2. CPL Materials Based on Liquid Crystal Polymers

### 2.1. LCP-Based CPL Materials Constructed by Covalently Grafting of Cholesterol Clusteroluminogens with Methacrylic Acids and Radical Polymerization

**Figure 2:**
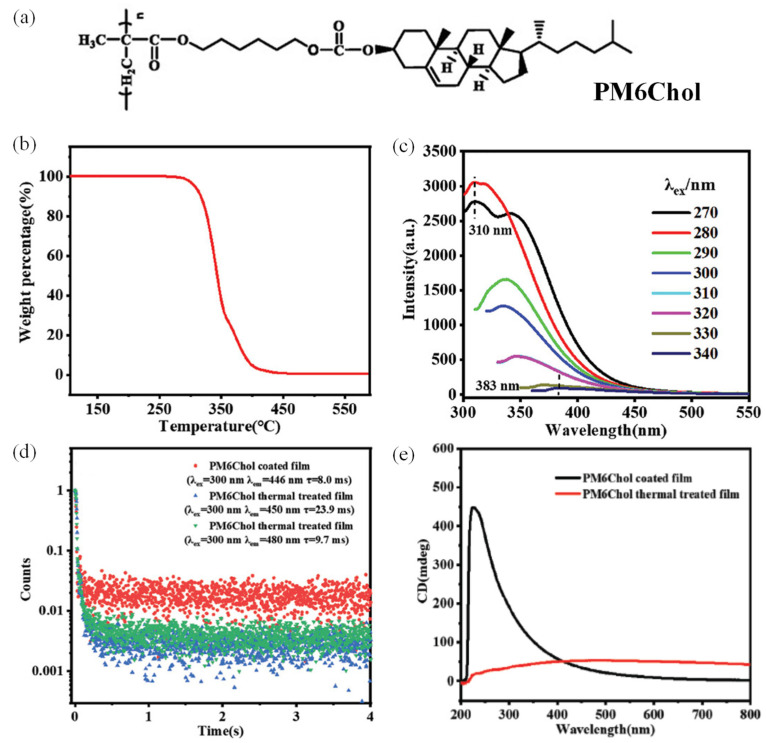
(**a**) Chemical structure of PM6Chol; (**b**) TGA curve of PM6Chol during the second heating process with a rate of 10 °C min^−1^; (**c**) emission spectra of PM6Chol in THF solutions with different excitation wavelength (2 mg mL^−1^); (**d**) RTP decay curves of PM6Chol-coated film and PM6Chol thermal-treated film; (**e**) CD spectra of PM6Chol-coated film and PM6Chol thermal-treated film. Reproduced with permission from [42], Wiley–VCH, 2023.

This strategy involves the design and synthesis of side-chain LCPs bearing cholesterol clusteroluminogens via chemical bonding. Taking advantage of liquid crystal phase features of cholesterol derivatives, distinct CPL properties can be obtained owing to their inherent molecular chirality. Yuan et al. [42] utilize covalent synthesis methods to link the cholesterol moiety and side-chain polymer groups through nucleophilic substitution reaction between methacrylic acid and cholesterol derivatives and radical polymerization. The synthesized LCP (PM6Chol) exhibited excellent thermal stability (decomposition temperature of 342 °C, 5% wt% loss) (Figure 2). The emission spectra for the PM6Chol-containing solutions exhibit excitation-wavelength-dependent luminescence characteristics, and the emission intensity of the solutions gradually decreases with increasing excitation wavelength, indicating typical clustering-triggered emissions [43]. In addition, PM6Chol-coated films show obvious afterglow characteristics with a long phosphorescence lifetime of 23.9 ms after they have been thermally treated. More intriguingly, PM6Chol films exhibit differentiated chiral optical absorption in that a strong positive cotton effect in its circular dichroism (CD) spectrum with an anisotropy factor of CD absorption of 1.1 × 10^−2^ for PM6Chol-coated film is observed. In comparison, for the PM6Chol thermal-treated film, this value changes to 5.2 × 10^−3^. This difference in CD spectra is because in the PM6Chol film, there is a bilayer chiral smectic C liquid crystal phase possessing helical structures, but after thermal treatment, the film forms a bilayer smectic A liquid crystal phase without helical structures. As for the CPL spectra, a *g*_lum_ value as high as 1.0 × 10^−1^ is obtained from the PM6Chol-coated film, while no CPL signal is observed from the PM6Chol thermal-treated film. Up to now, this value is the first one in the range of 10^−1^ for pure organic compound materials. Different from other works, the strategy adopted in this work indicates that constructing CPL materials based on single-component LCPs is feasible. This work provides a new perspective for the development of LCP-based CPL materials.

Yuan et al. [44] further demonstrate that copolymerization is also an effective method to obtain LCP systems with high-performance CPL. A chiral phosphorescent monomer M6Chol and a fluorescent monomer M6NI are covalently bonded and copolymer PNI-n containing different proportions of M6Chol and M6NI is obtained. It is found that the triplet–singlet Förstor-resonance energy transfer process can effectively improve the photoluminescent quantum yields of the copolymers. The highest |*g*_lum_| value for the copolymer could achieve 1.15. Anticounterfeiting and information security application by these copolymers are also explored. This work expands the range of polymer-based CPL materials. Different from the above work, Zheng et al. [45] also report LCP-based CPL materials utilizing main-chain LCPs via a chiral co-assembly strategy. The authors have synthesized three kinds of achiral LCPs that contain the emissive pyrenyl luminophore. After the incorporation of binaphthyl-based chiral dopants into the polymer, the composite system can assembly into helical nanofibers driven by the intermolecular π–π stacking interactions, showing weak CPL properties (*g*_lum_ = 0.015). Intriguingly, the CPL properties of the system become inverted and enlarged (*g*_lum_ = −0.064) after the thermal annealing process. This phenomenon is ascribed to the flexible main chain of the LCPs. This work provides a new perspective for the construction of CPL inversion materials and their related applications.

Since the four key elements in LCP materials are liquid crystals, polymers, chiral dopants, and luminescent sources, there are other kinds of covalent bond linked systems apart from the chemically connected systems between luminescent sources and liquid crystal polymers. Earlier than Yuan’s above works, Feng et al. [46] reported in 2020 one kind of designed and synthesized chiral polymer, poly (4-cholesterolformate-oxygen-tetraphenylethylene-methacrylate) (PT-Chol) that shows aggregation-induced emission properties. The authors combine the luminescent fragments and chiral polymer monomers chemically. The polymer is demonstrated to form a layered structure by polarized light microscopy and X-ray scattering characterization. After the introduction of liquid crystals 4-cyano-4′-pentyl biphenyl (5CB) into the polymer, the composite system shows efficient CPL properties with a *g*_lum_ value of ~+0.45. Further, it is demonstrated that with the proper concentration of PT-Chol in 5CB, chiral liquid crystals in smectic phase can come into formation, which results in highly efficient CPL properties of the system. This reported strategy of combining the luminophore and chiral polymer by covalent bonds via chemical synthesis provide new perspectives and guidelines for the design of LCP-based CPL materials. Later, Xie et al. [47] reported another chiral luminescent polymer in 2023, poly (4,4′,4″-tricholesterylformate-oxytetraphenylethylenemethyl acrylic acid ester) (P1). Although this polymer possesses chiral atoms and luminophore, no CPL signal is obtained for the polymer in the bulk. However, after the introduction of 5CB into the polymer, the system (P1@5CB) presents an obvious CPL behavior with a *g*_lum_ value of +0.18. Moreover, the chirality and Förster resonance energy transfer process are demonstrated from the polymer to the dye Nile red after the dye is introduced into the composite system. The energy transfer process gives rise to red CPL from Nile red with a *g*_lum_ value of +0.20. This work provides a new approach for the construction and modulation of polymer-based CPL properties. Another characteristic chiral luminescent polymer, a polyacetylene derivative, was reported by Zhao et al. [48], which plays a role of a chiral dopant and a polymer matrix. By doping the chiral luminescent polymer into the nematic liquid crystal of 5CB, a chiral nematic liquid crystal is established. A dissymmetry factor up to −1.87 is realized owing to the large helical twisting power of the polymer and the selective reflection effect of the liquid crystal. Furthermore, circularly polarized room-temperature phosphorescence with a *g*_lum_ value up to −1.57 is achieved by using a double-layered method. A chiral nematic liquid crystal-based anticounterfeiting application is also demonstrated, utilizing reflective color, chiroptical light, fluorescence, and circularly polarized fluorescence and room-temperature phosphorescence. This work opens up a new avenue for the polymer-based CPL materials and will stimulate the development of CPL materials with other photochemical and photophysical processes.

### 2.2. CPL Materials Constructed by Noncovalent Interactions Between LCPs and Luminescent Sources

#### 2.2.1. LCP-Based Hybrid Films Prepared by Host–Guest Co-Assembly of Achiral Dyes with LCPs for Controllable CPL

**Figure 3:**
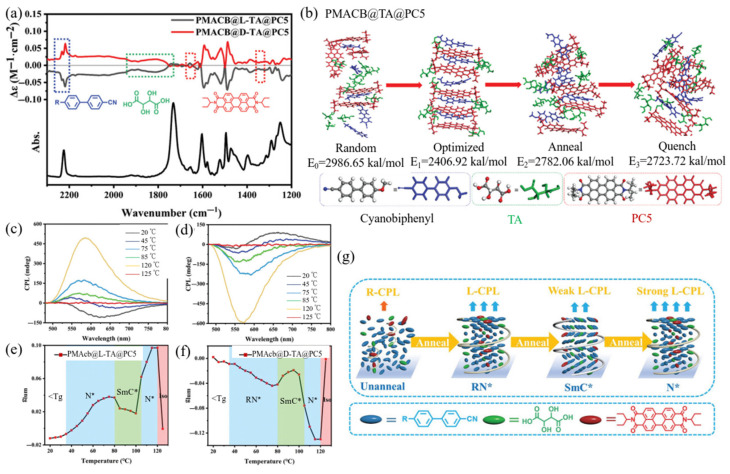
(**a**) Vibrational CD spectrum and corresponding Fourier transform infrared absorption spectrum of PMACB@TA@PC5 films; (**b**) energy-optimized structures by simulations; (**c**) temperature-dependent CPL spectra of PMACB@L-TA@PC5; (**d**) temperature-dependent CPL spectra of PMACB@D-TA@PC5; (**e**) temperature-dependent *g*_lum_ of PMACB@LTA@PC5; (**f**) temperature-dependent *g*_lum_ of PMACB@D-TA@PC5; (**g**) illustration of chiral amplification and inversion of the hybrid PMACB@L-TA@PC5 film. Reproduced with permission from [49], Wiley–VCH, 2024.

This strategy involves the material design and preparation methods of solid CPL films through host–guest co-assembly of LCPs and fluorescent dyes. Taking advantage of superior assembling features of LCPs, the incorporation of achiral fluorescent dyes into LCPs can favor the formation of regular arrangement of fluorescent molecules for enlarging the |*g*_lum_| value. Lu et al. [49] utilize one liquid crystalline polymer (polyacrylate bearing cyanobiphenyl mesogen side chains, PMACB) as a host material, enantiopure D-/L- tartaric acids (TA) as a chiral source guest, and achiral fluorescent N,N-bis (1-ethylpropyl)-perylene-3,4:9,10-bis (dicarboximide) (PC5) as a fluorescent probe guest in order to construct the hybrid films (Figure 3). The CD spectrum of the hybrid film proves that chirality transfer occurs from TA molecules to achiral PC5 and LCP. The vibrational circular dichroism spectra with specific peaks also verify that achiral PMACB segments and PC5 molecules produce chiral arrangement. In addition, the CPL and FT-IR tests suggest the existence of intermolecular interactions between PMACB (host) and TA (guest) and between PC5 and TA. Importantly, the calculation results by molecular dynamics also suggest that the intermolecular hydrogen bonding and π–π conjugation may be the driving force for the chirality transfer. The CPL spectra for the hybrid films show that the |*g*_lum_| value for the chiral nematic phase N* is the highest in four phases, in the range of 10^−1^ level. For such hybrid films, two origins for chiral CPL are proposed: the chiral arrangement of fluorescent molecules and the chiral liquid crystal phase. However, these two kinds of origins show an opposite effect, and the final chirality of CPL depends on the synergy effects of the two origins. In addition, the mass fraction of LCP in hybrid films can also affect the |*g*_lum_| value. When the PMACB fraction increases to 94%, the asymmetry factor shows a maximum of 0.30. This work proposes an efficient method of host–guest co-assembly of achiral fluorescent molecules, LCP, and chiral molecules for the construction of CPL films. This work provides a general way for the design and development of advanced chiroptical materials.

#### 2.2.2. High-Performance CPL Materials Prepared by Chirality–Induction Co-Assembly of Aggregate-Induced-Emission (AIE) Molecules with LCPs

**Figure 4:**
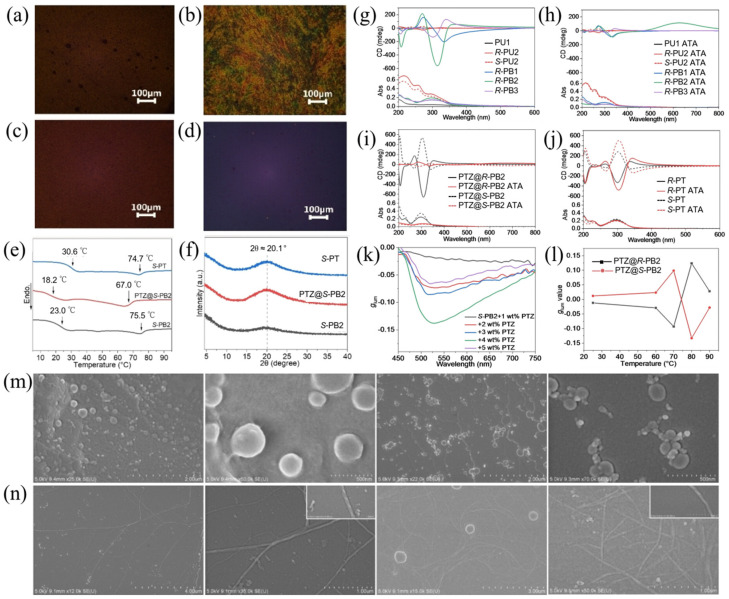
(**a**) The polarizing optical microscope (POM) image of PTZ@*S*-PB2 at 66 °C in the heating state; (**b**) the POM image of *S*-PT at 70 °C in the heating state; (**c**) the POM image of PTZ@*S*-PB2 at room temperature after thermal annealing at 80 °C; (**d**) the POM image of *S*-PT at room temperature after thermal annealing at 80 °C; (**e**) DSC second heating curves of *S*-PT, PTZ@*S*-PB2, and *S*-PT; (**f**) XRD patterns of *S*-PT, PTZ@*S*-PB2, and *S*-PT; (**g**) absorption and CD spectra of PU1, *R/S*-PU2, and *R*-PB1/2/3 in spin-coated films; (**h**) absorption and CD spectra of PU1, *R/S*-PU2, and *R*-PB1/2/3 in spin-coated films after thermal annealing at 80 °C; (**i**) absorption and CD spectra of PTZ@*R/S*-PB2 and *R/S*-PT; (**j**) absorption and CD spectra of PTZ@*R/S*-PB2 and *R/S*-PT after thermal annealing at 80 °C; (**k**) *g*_lum_ values of *S*-PB2 doped with different wt% of PTZ in the spin-coated films after thermal annealing at 80 °C; (**l**) *g*_lum_ values of PTZ@*R/S*-PB2 (*λ*_em_ = 550 nm) after thermal annealing at different temperatures; (**m**) SEM images of LCPs of PTZ@*S*-PB2 (left two panels) and *S*-PT (right two panels) before thermal annealing; (**n**) SEM images of LCPs of PTZ@*S*-PB2 (left two panels) and *S*-PT (right two panels) after thermal annealing at 80 °C (films, 10^−1^ mg/mL in chlorobenzene). Reproduced with permission from [50], Wiley–VCH, 2024.

This strategy involves the chiral co-assembly strategy to incorporate the AIE molecules with LCPs for high-performance CPL. Taking advantage of the good chirality-induction ability of chiral binaphthyl units with anchored dihedral angles, ternary chiral co-assembled films composed of chiral binaphthyl-containing liquid crystal polymers and achiral dye can exhibit AIE-active characteristics and high-performance CPL properties. Cheng et al. [50] utilize achiral mesogenic monomer (M1), chiral binaphthyl monomer (R/S-M2), and achiral dye (PTZ) to construct ternary co-assembled LCP systems through radical polymerization reactions (Figure 4). Clear nematic LC textures of ternary hybrid systems captured by polarized optical microscope and X-ray diffraction (XRD) patterns confirm the formation of LC phases. The CD spectrum of unary, binary, and ternary polymers show evident asymmetric absorption features. The CPL intensity of ternary systems show a relatively high |*g*_lum_| value of 0.14 with a PTZ doping ratio of 4 wt%. The helical nanofiber structures are believed to be the origin of the amplified CPL emissions. Moreover, the effect of the film thickness on CPL performance is investigated; the maximum |*g*_lum_| of the ternary film reaches up to 0.91 when the thickness of the film is 220 nm. This work develops a crucial method of chiral co-assembly to construct high-performance CPL-active materials.

The Cheng group [51,52] has devoted itself to the development of liquid crystal-based CPL systems in recent years. For instance, this group reported an aggregation-induced emission-active chiral fluorescence emitters for the combination with nematic liquid crystals to produce high-performance CPL. Owing to the dipolar interactions between the emitters and the host nematic liquid crystals, the prepared systems show a large dissymmetry factor *g*_lum_ up to 0.41. More recently, this group reported achiral dichroic dye-mediated CPL in cholesteric liquid crystals. The relationship between the orientational order parameter (*S*_F_) of achiral dyes and cholesteric liquid crystals is investigated, demonstrating that the larger the *S*_F_ value (0.3), the stronger the CPL signal (|*g*_lum_| = 0.41).

In 2022, Liu et al. [53] reported on the polymer-stabilized cholesteric liquid crystal films prepared by the same co-assembly strategy. The authors synthesized an achiral dye molecule of tetraphenylethene and imidazeo [1,2-α]pyridine-attached difluoroboronβ-diketonate triad (TPE-BF_2_acac-MeIP) and introduced it into the polymer-stabilized cholesteric liquid crystal systems. Due to the good aggregation-induced emission and mechanofluochromism properties of TPE-BF_2_acac-MeIP, the composite systems show high emission quantum yield (64.2%) and excellent CPL properties (|*g*_lum_| = 0.61). The large |*g*_lum_| is attributed to the good matching between the emission of the dye and the selective Bragg reflection wavebands of the LCPs. This work offers researchers in the CPL material field new understanding and insight into the origin of CPL behavior. Yang et al. [54] reported a double-LCP based CPL system using a similar chiral co-assembly strategy. The authors adopted double LCP monomers and aggregation-induced emission-active luminogens and combined them into liquid crystalline polymers. A dissymmetry factor of up to 0.71 was also achieved.

#### 2.2.3. Perovskite Quantum Dots–LCPs Composites as Flexible and Stable CPL Materials Prepared by a Double-Layer Construction Strategy

**Figure 5:**
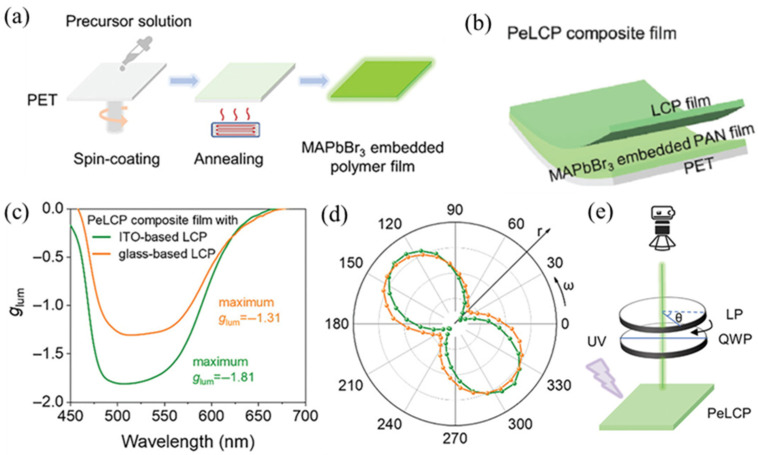
(**a**) Schematic illustration of the fabrication process of perovskite-embedded polymer films on a flexible substrate; (**b**) schematic illustration of a PeLCP composite film; (**c**) the *g*_lum_ value plots of *L*-PeLCP composite films; (**d**) emission intensity of *L*-PeLCP composite films containing ITO glass-based (green line) and glass-based LCP films (orange line) as a function of polarization angle in the polar coordinate system; (**e**) schematic illustration of the variation in luminescence intensity of the patterned PeLCP with stacked multilayer films with a QWP and a linear polarizer by rotating different angles under ultraviolet (UV) light irradiation. Reproduced with permission from [55], Wiley–VCH, 2024.

This strategy involves a robust construction strategy for the preparation of flexible CPL films. The flexible CPL films consist of a double-layer structure: one layer of polymer film embedded with perovskite nanocrystals and another layer of LCP film. Taking advantage of the luminescence feature of perovskites and flexibility characteristics of LCPs, the double-layered films can combine advantages of both materials simultaneously. Wu et al. [55] utilized a polyethylene terephthalate (PET) film as a flexible substrate, one perovskite (PEABr and MABr) nanocrystal-embedded polyacrylonitrile (PAN) film as an emission layer, and a liquid crystal polymer film as a chiral light selection layer to construct flexible CPL film composites (Figure 5). The composite film shows a maximum |*g*_lum_| value of 1.81, which is the highest value for flexible CPL films so far. Moreover, the composite films exhibit excellent mechanical and environmental robustness, with the |*g*_lum_| value remaining larger than 1.6 after 21 days under water exposure, heating at 80 °C, or after 1000 bending cycles. Upon a mask template-controlled UV photopolymerization method, the composite films were applied as anti-counterfeiting labels. This work extends the boundary of the field of CPL materials research and opens up a new avenue for advanced optoelectronic applications.

#### 2.2.4. Full-Color CPL Materials Constructed by Combining Chiral Helical Polymer and Liquid Crystal Monomers via Photopolymerization

**Figure 6:**
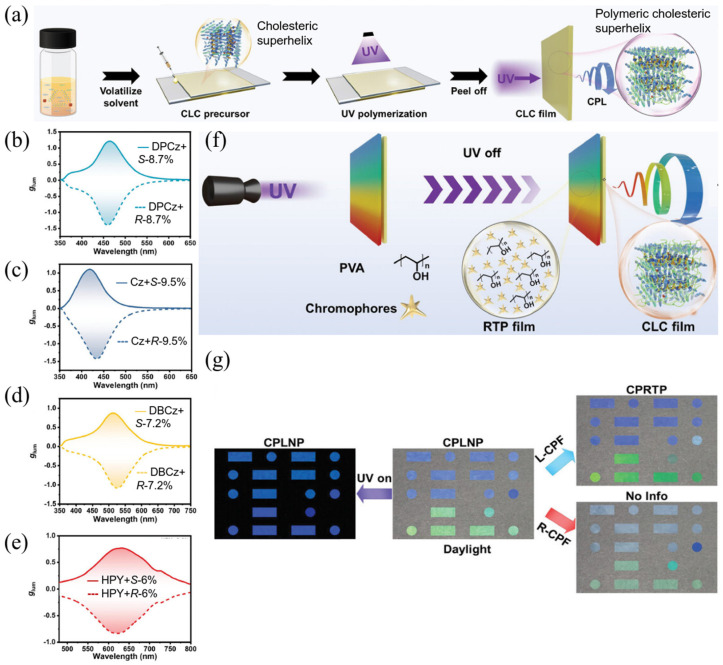
(**a**) Detailed process diagram for the preparation of LC films; (**b**) *g*_lum_ spectra of double-layered circularly polarized RTP system including DPCz-doped PVA film and CLC films; (**c**) *g*_lum_ spectra of double-layered circularly polarized RTP system including Cz-doped PVA film and LC films; (**d**) *g*_lum_ spectra of double-layered CPRTP system including DBCz-doped PVA film and LC films; (**e**) *g*_lum_ spectra of double-layered circularly polarized RTP system including HPY-doped PVA film and LC films; (**f**) illustration of full-color circularly polarized RTP based on double-layered films consisting of RTP film and LC film; (**g**) experimental demonstration of the Morse code information scenario utilizing structural color, fluorescence, RTP, and circularly polarized RTP of the double-layered films. Reproduced with permission from [56], Wiley–VCH, 2024.

This strategy features the incorporation of nematic liquid crystals with polymers bearing chiral helical superstructures. Taking advantage of chiral helical superstructures of polymers, combined polymeric films can exhibit excellent CPL properties. Zhao et al. [56] proposed a universal strategy to fabricate high-performance CPL materials by photopolymerizing mixtures of nematic liquid crystal monomers and a chiral helical polymer (CHP) (Figure 6). This study marks the first instance where chiral helical polymers function as chiral dopants to construct cholesteric superhelices. The largest |*g*_lum_| value obtained from the as-fabricated LCPs is as high as 1.45 when the liquid crystal monomer was doped with 8.9 wt% *R*-CHP. Furthermore, a facile strategy combining LCPs with room-temperature phosphorescence (RTP) films in double-layer architectures was proposed to achieve high-performance circularly polarized RTP. The RTP film, composed of polyvinyl alcohol (PVA) doped with 3,6-diphenylcarbazole, is covered by an LCP film acting as a chiral filter. When the RTP film is excited by the UV light, the emission from this film coincides with the selective reflection band of the LCP film, thus producing the circularly polarized RTP. The phosphorescence lifetime of the double-layered film is 2431.29 ms, and its |*g*_lum_| value of circularly polarized RTP is as high as 1.22 at 465 nm. Furthermore, by changing the doped material (carbazole, 7H-dibenzo [c,g]-carbazole, and 1-hydroxypyrene) in PVA films, the CPL emission covers the full spectrum from blue to green and yellow wavebands, with |*g*_lum_| values of 1.43 at 437 nm, 1.09 at 515 nm, 0.84 at 625 nm, respectively. Taking advantage of the distinct fluorescence, RTP, and circularly polarized RTP properties of double-layered structures, multilevel information encryption applications based on the digital pattern and Morse code are carried out. This work successfully constructed full-color circularly polarized RTP materials consisting of double-layered (phosphorescent film and LCP film) architectures. More importantly, the concept of circularly polarized RTP from double-layered structures is proposed, thus providing a new strategy for the construction of chiral RTP materials and chiroptical devices.

### 2.3. Other CPL Hybrid Systems Containing LCs or/and LCPs for Future Possible CPL-Based Applications

**Figure 7:**
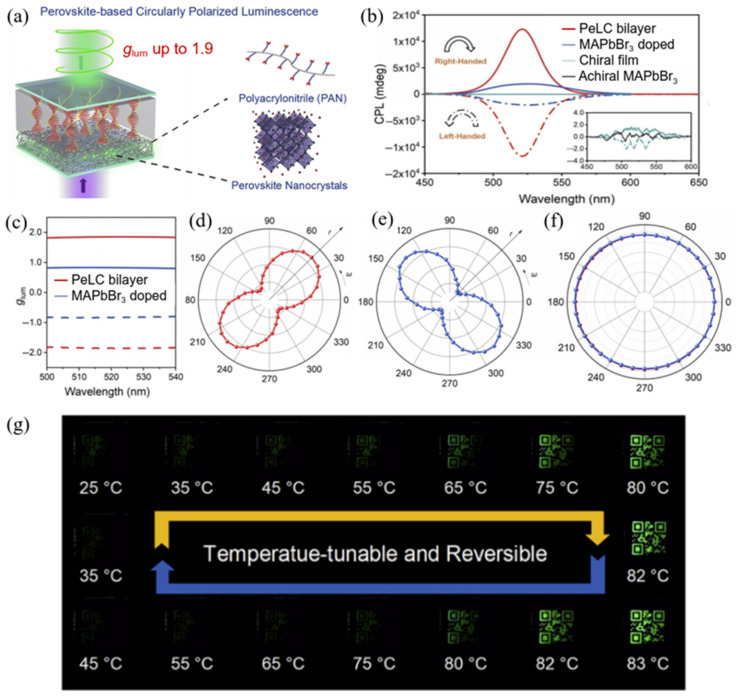
(**a**) Configuration of the perovskite-liquid crystal (PeLC) bilayer-featured device; (**b**) CPL spectra of PeLC bilayer device, MAPbBr_3_ QD-doped chiral LC device, chiral (*R*- or *S*-MBA)_2_MAPb_2_Br_7_ film, and achiral MAPbBr_3_ film; (**c**) variation in g_lum_ values against wavelength of PeLC bilayer device and chiral LC device; (**d**–**f**) emission intensity of left-handed (**d**) and right-handed PeLC bilayer devices as a function of polarization angle with (**d**,**e**) and without (**f**) the quarter wavelength plate in the polar coordinate system; (**g**) photographs of the PeLC bilayer device loaded with a quick response code as a function of temperature under UV light irradiation (365 nm). Reproduced with permission from [57], Elsevier, 2022.

In this section, distinctive strategies beyond the ones discussed above for constructing high-performance LC/LCP-based CPL materials and exploring their applications are selectively introduced. Different from conventional construction strategies for single-layer multicomponent hybrid CPL films, the strategy of constructing composite systems with bilayer architectures was proposed and carried out by Zhu et al. [57] in 2022 (Figure 7). This strategy involves the design and preparation of perovskite-incorporated polymer–liquid crystal double-layered hybrid CPL materials. Perovskite-incorporated polymer films exhibit excellent photoluminescence quantum yield (PLQY) (97.5%, MAPbBr_3_ quantum dots in polyacrylonitrile). More importantly, perovskite polymer–chiral liquid crystal double-layered hybrid films show excellent CPL performance (|*g*_lum_| = 1.9). This performance is much better than that of perovskite quantum dots (QDs)-doped chiral LC devices (|*g*_lum_| = 0.84), which is ascribed to the better coupling effect between perovskite and chiral LC in the double-layered devices. Furthermore, anti-counterfeiting scenario based on thermal-switchable CPL bilayer devices is carried out. This work demonstrates the feasibility of obtaining a large |*g*_lum_| value and high PLQY simultaneously. In addition, Zhu et al. [58] recently published a perspective article on liquid crystal assembly for ultra-dissymmetric CPL and beyond. This perspective article provides a solid theoretical foundation for the development of liquid crystal-based CPL systems.

Liu et al. [59] recently reported a cooperative noncovalent–covalent strategy to amplify CPL in chiral liquid crystals. By covalently anchoring an anthraquinone chromophore to the diarylacetylene component of LCs via a photoinduced addition reaction, the liquid crystal composites are finally fabricated and an unprecedented *g*_lum_ value of up to 1.73 is achieved. Furthermore, these composites were demonstrated in a Morse code-based multiple information encryption scenario.

**Figure 8:**
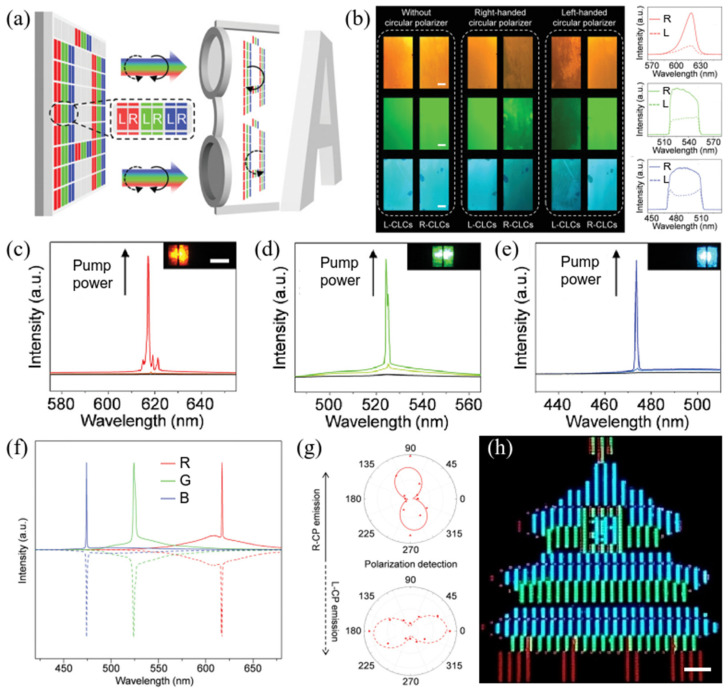
(**a**) Schematic illustration of 3D laser displays based on CP microlaser arrays; (**b**) fluorescence images (left panels) and PL spectra (right panel) of R-CLC and L-CLC cells doped with red-, green-, and blue-emissive dyes; (**c**–**e**) PL spectra of red-, green-, and blue-emissive CLC microunits under different pump fluences; (**f**) RGB circularly polarized lasing spectra from right-CLC microunits (solid line) and left-CLC microunits (dashed line); (**g**) right and left circularly polarized laser emission intensity plots as a function of the polarization angle from the red-emissive right-CLC and left-CLC microunits, respectively; (**h**) image of 2.0 cm (diagonal) laser display prototype using as-fabricated panel with a 27 × 27 pixel array. Scale bar: 1 mm. Reproduced with permission from [60], Wiley–VCH, 2021.

In order to further explore the application potential of CPL materials, Yan et al. [60] reported a strategy of utilizing chiral LC materials to construct pixelated red–green–blue (RGB) microlaser arrays that can emit bright CPL and demonstrate proof-of-concept full-color three-dimensional (3D) laser displays (Figure 8). Cholesteric LCs (CLCs) with specific chirality exhibit significant selective reflection characteristics, e.g., LCs with right-handed helix reflect right-CPL much more evidently than left-CPL. Through ultrasonic-assisted inkjet printing, dye-doped cholesteric LCs serving as inks are injected into orderly packed polymer templates, giving rise to LC microlaser pixel arrays as display panels. In an individual pixel, there are two sets of RGB-emissive subpixels filled with left-handed and right-handed dye-doped LCs, respectively. With increasing pump power, the RGB-emissive pixels show narrow-bandwidth single-mode lasing behavior. Right-handed and left-handed circularly polarized emissions from the subpixels are characterized and verified by the polarization detection via a quarter wave plate and a polarizer. Moreover, the emission from differently combined RGB-emissive pixels shows an excellent color rendering effect, presenting vivid mixed colors such as yellow, cyan, magenta, and white in the far field. On the basis of full-color displays, a concept demonstration of 3D laser displays is carried out. This work provides a new construction strategy for 3D display panels and verifies the potential of chiral LC materials on 3D displays.

## 3. Conclusions

In this review, we summarize the development of LCP-based CPL materials over the past decade. Regarding design strategies, approaches such as self-assembly, host–guest systems, co-assembly, and double-layered LCP materials have been employed to construct high-performance CPL systems via incorporation of organic dyes, quantum dots, polymers, and so on. More importantly, the mesoscopic chiral environment of LCPs, arising from their helical superstructures, imparts relatively high luminescence chirality to the emissive sources. Thus, LCP composite systems exhibit excellent chirality transfer and chiral luminescence performance. Overall, LCP composite systems combine the chiral properties of liquid crystals and the good luminescence characteristics of luminous molecules or units, giving rise to large |*g*_lum_| values ultimately. Additionally, these materials show promising applications in the field of information encryption and 3D displays. Although LCP materials show outstanding advantages especially on the construction of CPL materials, this kind of materials also exhibit limitations in the compatibility with some kinds of luminous sources, for example, inorganic quantum dots. This incompatibility not only affects the photoluminescence quantum yields of LCP composites, but it also limits their practical applications in 3D displays, information encryption, and other scenarios. The incompatibility also contributes to the relatively low |*g*_lum_| values (in the range of 10^−2^ to 10^−1^) for most of LCP-based CPL materials. In addition, the chirality enhancement mechanism of the systems induced by the LCP chiral environments is still unclear. This problem impedes further improvements in the CPL performance of LCP-based composite systems.

Building on the summarized development status of LCP-based CPL materials, we present an outlook on future research directions, particularly emphasizing emerging applications. Taking advantage of the unique polarized emission features, LCP-based CPL materials hold strong potential for use in information safety (e.g., encryption and anticounterfeiting) and display technologies (e.g., 3D displays and virtual reality) scopes. Moreover, LCP-based CPL materials hold great promise for next-generation applications, including high-contrast bioimaging and smart sensors, efficient optoelectronic devices and lasers, interactive smart textiles, quantum computing and high-density data storage, as well as enantioselective photocatalysis and spintronic devices, and other cutting-edge scientific and technological fields. This review is expected to not only provide deep insights into the fundamentals of chirality transfer but also to present new development routes for advanced chiral luminescent materials, further exploring their promising applications in the fields of chiral photonics such as quantum communication, optical information processing, spintronics, and beyond.
